# Data Resource Profile: STHLM0, the Stockholm Prostate Cancer Diagnostics Register

**DOI:** 10.1093/ije/dyaf062

**Published:** 2025-06-03

**Authors:** Ahmad Abbadi, Martin Eklund, Markus Aly, Mark Clements, Alessio Crippa, Andrea Discacciati, Astrid Björklund, Vivekananda Lanka, Chiara Micoli, Anna Lantz, Henrik Grönberg, Tobias Nordström

**Affiliations:** Department of Medical Epidemiology and Biostatistics, Karolinska Institutet, Solna, Sweden; Department of Medical Epidemiology and Biostatistics, Karolinska Institutet, Solna, Sweden; Department of Molecular Medicine and Surgery (Solna), Karolinska Institutet, Stockholm, Sweden; Department of Medical Epidemiology and Biostatistics, Karolinska Institutet, Solna, Sweden; Department of Medical Epidemiology and Biostatistics, Karolinska Institutet, Solna, Sweden; Department of Medical Epidemiology and Biostatistics, Karolinska Institutet, Solna, Sweden; Department of Medical Epidemiology and Biostatistics, Karolinska Institutet, Solna, Sweden; Department of Medical Epidemiology and Biostatistics, Karolinska Institutet, Solna, Sweden; Department of Medical Epidemiology and Biostatistics, Karolinska Institutet, Solna, Sweden; Department of Medical Epidemiology and Biostatistics, Karolinska Institutet, Solna, Sweden; Department of Molecular Medicine and Surgery (Solna), Karolinska Institutet, Stockholm, Sweden; Department of Medical Epidemiology and Biostatistics, Karolinska Institutet, Solna, Sweden; Department of Oncology, Capio St Görans Sjukhus, Stockholm, Sweden; Department of Medical Epidemiology and Biostatistics, Karolinska Institutet, Solna, Sweden; Department of Clinical Sciences at Danderyds Hospital, Karolinska Institutet, Solna, Sweden

**Keywords:** prostate cancer, prostate-specific antigen, magnetic resonance imaging, cancer screening, biomarkers

Key Features

- STHLM0, the Stockholm Prostate Cancer Diagnostics Register, is a comprehensive, population-based registry that includes all men who have undergone prostate cancer diagnostic testing in Stockholm County since 2003, regardless of the test(s) outcomes.
- The primary aim of STHLM0 is to gather comprehensive data on all men tested for prostate cancer in the Stockholm County. Data are sourced from all laboratories across the county. Once registered, men are followed up until death or emigration, and data are supplemented by linking relevant information from Swedish National Health and Population registers, including those maintained by the National Board of Health and Welfare, and the National Prostate Cancer Register. Linked data include, but are not limited to, demographic information, access to specialized outpatient clinics, hospitalizations, cancer diagnoses, causes of death, prescribed medications, and detailed prostate cancer clinical data. The registry is updated annually as of December 2024.
- Since 2003, a total of 550 778 men have tested for prostate cancer and are included in the register (as of the latest update on 31 December 2021). STHLM0 offers a unique resource for epidemiological research, providing valuable insights into prostate cancer testing, treatment modalities, associated adverse events, morbidities, and mortality.
- Collaborative research efforts are encouraged and further information is available at https://ki.se/en/research/research-areas-centres-and-networks/research-groups/henrik-gronbergs-research-group#tab-sthlm0.

## Data resource basics

### Background

Prostate cancer is the second-most common cancer among men worldwide [1] and the most common cancer among men in Sweden [2]. In 2022, prostate cancer accounted for 31.0% of incident cancers and 18.7% of all cancer-related deaths among Swedish men, making it the primary cause of cancer mortality in this population [2].

The primary goal of prostate cancer screening is to detect the disease at an early stage, when it is more likely to be curable [3]. However, current medical guidelines do not recommend national prostate cancer screening programs [4, 5]. Instead, they emphasize the importance of individual-level testing after discussions with the patient through shared decision-making [4, 5]. Nonetheless, it remains crucial to document testing outcomes and assess their impact on health and mortality to inform robust epidemiological and clinical guidelines [6].

Sweden benefits from well-established registries that leverage personal identification numbers, enabling the linkage of data across various sectors [7]. While the National Prostate Cancer Register (NPCR) gathers comprehensive data on prostate cancer cases in Sweden, including clinical characteristics, treatments, and outcomes [8], it does not include men who were tested for prostate cancer and received negative results. Furthermore, information on prostate-specific antigen (PSA) levels and prostate biopsies is not captured unless the results are positive, with no documentation of historical PSA results pre-diagnosis [8].

To address these gaps, STHLM0—the Stockholm Prostate Cancer Diagnostics Register (STHLM0 pronounced “Stockholm noll” or “Stockholm zero”) was established [9, 10]. STHLM0 aims to encompass all men tested for prostate cancer in Stockholm County (Stockholm län), regardless of the outcome, and provides the data on PSA results, biopsies performed, Stockholm3 test, and MRI. This broader population base offers critical information that can support randomized–controlled trials and other prostate cancer-related studies, with valuable exposure, confounders, and outcome data.

In addition to the unique dataset, STHLM0 also integrates existing data from several national registers. However, it does not include data from primary healthcare, but with plans for expansion.

This database resource profile introduces STHLM0, outlining the challenges faced during data collection, the advances and progress of the register over time, its usage in research, and future directions. STHLM0 is supported by funding from various governmental bodies and foundations for projects conducted using this register, ensuring its ongoing management and expansion.

Information on the method of constructing this study is available in Supplementary File 1.

### The STHLM0 register

STHLM0 is a population- and register-based cohort. Data are collected by using the Swedish personal identification number (personnummer) of 12 digits that is unique to each individual living in Sweden [7]. STHLM0 was developed in 2008/2009 and formally established in 2012 under the name of the Stockholm PSA and Biopsy Register (STHLM0), with collected data from the three Stockholm County-based laboratories [Karolinska University Laboratory (KUL), Synlab (formerly known as Aleris), and Unilabs] that have performed PSA testing since 2003 [9, 10]. The same laboratories have pathology units that process and provide the reports on prostate biopsies; an additional fourth laboratory also reports on biopsies (Inify) [9, 10]. Auxiliary diagnostic tests, such as the Stockholm3 test, are performed at A3P Biomedical and results are reported to STHLM0 [11]. The register currently includes all the laboratories performing prostate cancer diagnostics in Stockholm County and any newly established laboratory is reached out to for inclusion in the register. In 2024, the register was renamed “STHLM0: the Stockholm Prostate Cancer Diagnostics Register” to reflect its inclusivity to a large domain of variables that go beyond two diagnostic tests and include clinical follow-ups and health outcomes.

### Inclusion in the STHLM0

All men tested for prostate cancer by using PSA, Stockholm3 test, or MRI, or have done a prostate biopsy in the Stockholm County are included in the register. Like other Swedish registers, the inclusion is automatic, but the participants can opt out in accordance with Swedish laws and regulations. There are no limitations regarding age or test outcomes for inclusion.

### Characteristics of participants

**Figure 1.**
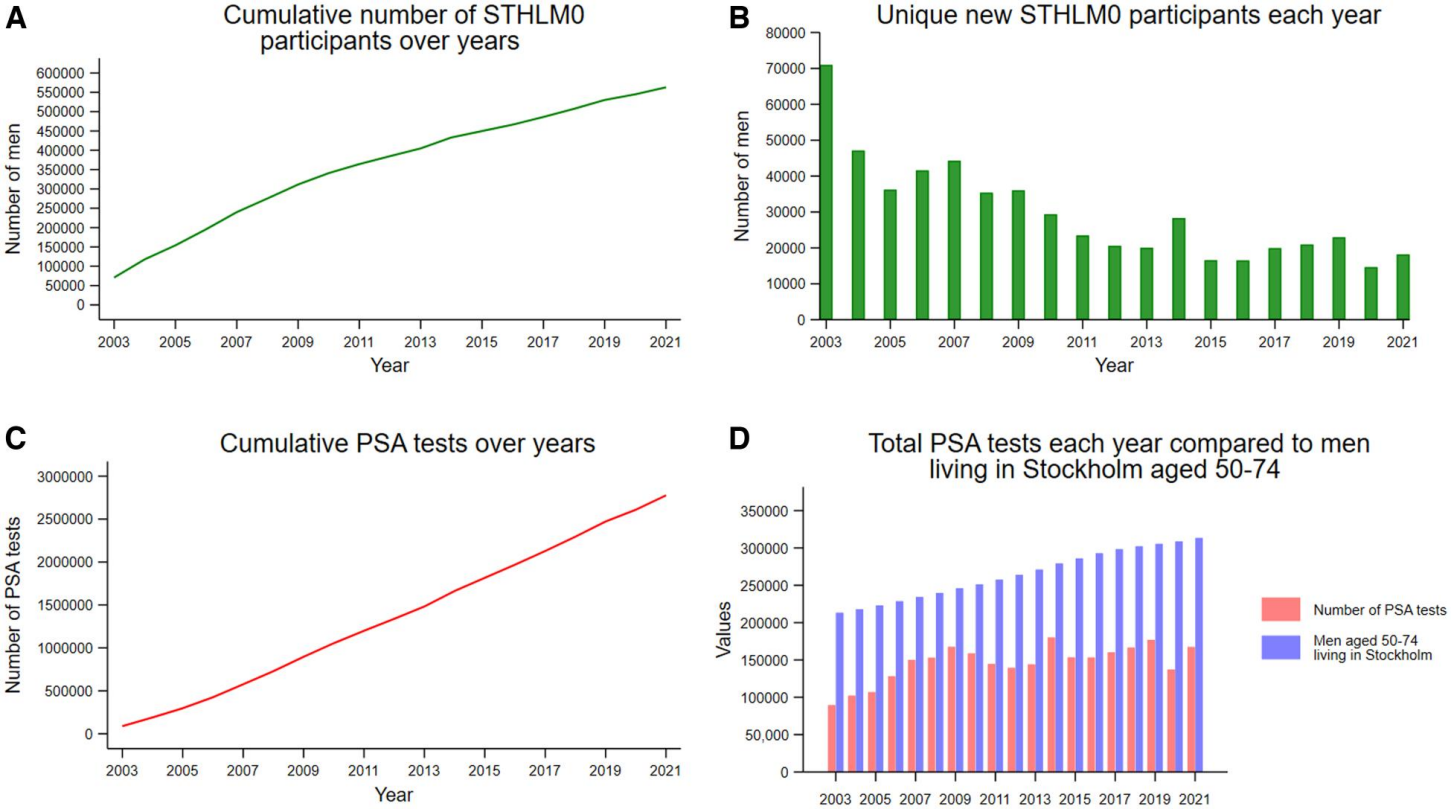
Per-year outcomes in Stockholm County from 2003 to 2021. (A) Cumulative number of STHLM0 participants. (B) Unique new participants included in STHLM0. (C) Cumulative PSA tests performed. (D) Contrasting the number of PSA tests performed each year in Stockholm to the target group (men aged 50–74 years) per Swedish guidelines.

Based on the latest update (31 December 2021), the clinical characteristics and demographic information of all men included in STHLM0 can be found in Table 1. Overall, 550 778 men have been included in the registry since 2003. In total, 2 777 183 PSA tests were recorded. Figure 1A shows the cumulative number of men included in STHLM0, while Figure 1B shows the number of men included in STHLM0 each year (new participants). Figure 1C shows the cumulative number of PSA tests performed in the Stockholm County and Figure 1D shows the number of PSA tests done in each year contrasted with the number of men aged 50–74 years living in Stockholm County in that year.

**Table 1.** Demographic and clinical characteristics

Variable	Men living in Stockholm County and their first-degree relatives (*n *=* *2 267 103)	Tested for prostate cancer (*n *=* *550 778)	Diagnosed with prostate cancer (*n *=* *45 746)
PSA test [*n* (%)]			
No	1 716 878 (75.7%)	558 (0.1%)	82 (0.2%)
Yes	550 225 (24.3%)	550 225 (99.9%)	45 664 (99.8%)
Biopsy [*n* (%)]			
No	2 200 219 (97.0%)	483 894 (87.9%)	10 645 (23.3%)
Yes	66 884 (3.0%)	66 884 (12.1%)	35 101 (76.7%)
Age in years at first prostate cancer diagnostic [median (IQR)]	56.5 (49.0, 65.1)	56.5 (49.0, 65.1)	64.9 (58.5, 73.1)
Age in years at first prostate cancer diagnostic [*n* (%)]			
<50	155 549 (6.9%)	155 549 (28.3%)	2344 (5.1%)
50–59	181 157 (8.0%)	181 157 (32.9%)	11 765 (25.7%)
60–69	125 550 (5.5%)	125 550 (22.8%)	16 583 (36.3%)
70–79	59 069 (2.6%)	59 069 (10.7%)	10 078 (22.0%)
80+	28 747 (1.3%)	28 747 (5.2%)	4972 (10.9%)
Not tested	1 716 325 (75.7%)	0	0
Educational level at first PSA test [*n* (%)]			
Primary school or ≤9 years of formal education	99 387 (4.4%)	99 387 (18.0%)	9004 (19.7%)
High school or between 10 and 12 years of formal education	220 371 (9.7%)	220 371 (40.0%)	17 288 (37.8%)
College or higher, >12 years of formal education	210 962 (9.3%)	210 962 (38.3%)	15 946 (34.9%)
Missing	20 058 (0.9%)	20 058 (3.6%)	3508 (7.7%)
Not tested	1 716 325 (75.7%)	0	0
Civil status at first PSA test [*n* (%)]			
Not married	116 163 (5.1%)	116 163 (21.1%)	5713 (12.5%)
Married	326 915 (14.4%)	326 915 (59.4%)	29 548 (64.6%)
Divorced	86 835 (3.8%)	86 835 (15.8%)	7340 (16.0%)
Widowed	18 925 (0.8%)	18 925 (3.4%)	2841 (6.2%)
Missing	1940 (0.0%)	1940 (0.4%)	304 (0.7%)
Not tested	1 716 325 (75.7%)	0	0
Result of first PSA test in ng/mL [median (IQR)]	1.0 (0.6, 2.0)	1.0 (0.6, 2.0)	3.9 (1.8, 9.2)
Lab reading error at first PSA [*n* (%)]	14 809 (0.6%)	14 809 (2.7%)	2467 (5.4%)
Not tested with PSA	1 716 878 (75.7%)	558 (0.1%)	82 (0.2%)
PSA retesting^a^ [*n* (%)]			
No	124 820 (5.5%)	124 820 (22.7%)	643 (1.4%)
Yes	425 958 (18.8%)	425 958 (77.3%)	45 103 (98.6%)
Not tested with PSA	1 716 878 (75.7%)	558 (0.1%)	82 (0.2%)
Number of PSA tests performed^b^ [median (IQR)]	2.0 (1.0, 6.0)	2.0 (1.0, 6.0)	15.0 (9.0, 22.0)
Not tested with PSA	1 716 878 (75.7%)	558 (0.1%)	82 (0.2%)
Prostate cancer [*n* (%)]			
No	505 032 (22.3%)	505 032 (91.7%)	0
Yes	45 746 (2.0%)	45 746 (8.3%)	45 746 (100%)
Not tested	1 716 878 (75.7%)	0	0
Death during follow-up [*n* (%)]			
No	1 818 722 (80.2%)	445 206 (80.8%)	29 130 (63.7%)
Yes	448 381 (19.8%)	105 572 (19.2%)	16 616 (36.3%)

ng, nanogram; mL, milliliter; IQR, interquartile range; PSA, prostate-specific antigen.

a PSA retesting refers to men who had more than one PSA test during the follow-up of STHLM0.

b Number of PSA tests performed refers to the median number of PSA tests conducted by men during the entire follow-up of STHLM0 from January 2003 to December 2021.

### Follow-up

Once a man is included in the study, he will be followed up until death or emigration. However, there are no specific intervals for follow-up, as the information is collected continuously based on the laboratories and the registers linked. As of 2024, STHLM0 was updated annually in December of each year. The register is intended to continue collecting data with no end date.

At each update cycle, STHLM0 is updated to 31 December of the previous year. Moreover, there is an additional 1-year delay in reporting the cause of death, but not the date of death.

### Ethical clearance

STHLM0 was approved by the local ethics committee at Karolinska Institutet and regional ethics board in Stockholm. Detailed ethics approvals and amendments to the applications are summarized in Supplementary Table S1.

## Data collected

### Primary data unique to STHLM0

Data on prostate cancer diagnostics are unique to STHLM0 and are not available in other quality registers unless they resulted in diagnosis. The data include PSA, prostate biopsy (including Gleason grading), MRIs, and Stockholm3 tests reported from the laboratories covering the Stockholm County. As of 2024, the reporting from each laboratory was performed quarterly. The reporting from the laboratories was done at the Swedish personal identification number level and was then sent to Statistics Sweden (SCB) for pseudonymization and linkage to the other registers.

### Linkages to other registers

Once a person is included in STHLM0 and diagnostics results from the labs are reported, additional data are collected from other Swedish registers. All the Swedish registers used for linkage have high reported completion and validity [12–15]:

- Swedish Population Register.
  This register is managed by the Swedish Tax Agency and delivers its data to Statistics Sweden that organizes it under Total Population Register [12]. Data on the date of birth, date of (im/e)migration, municipality, civil status, country of birth, and death are collected and linked to STHLM0.
- The Longitudinal Integrated Database for Health Insurance and Labour Market Studies (LISA).
  This register was established in 2003, but includes data since 1990 [16]. The register has data on education, income from employment, capital or allowances, unemployment, sick leave, and disability pensions [16]. Data on education and income are available for each year.
- National Cancer Register.
  This register is managed by the National Board of Health and Welfare, and has recorded cancer cases in Sweden since 1958 [17]. It is regulated by law, as healthcare workers are obliged to report diagnosed cancer cases [17]. Diagnoses of prostate cancer and other cancers are linked to STHLM0. Data are available on the date of diagnosis, diagnosis code, and tumor, node, metastasis staging system (TNM) stage.
- National Patient Register.
  This register includes data on both inpatient and outpatient specialized care with diagnoses using International Classification of Disease-10 (ICD-10) codes for patients attending healthcare at secondary and tertiary settings [18]. The inpatient register was established in 1964, with national coverage since 1987, and includes all hospitalizations, while the outpatient register was established in 2001 and includes specialized outpatient clinic visits (excludes primary care outpatient visits) [18]. Date of healthcare visit, age at the visit, reason for visit, diagnoses, medical procedures, discharge (for hospitalizations), and location of health service are linked to STHLM0.
- Cause of Death Register.
  This register includes all deaths since 1952 for residents in Sweden and nonresidents since 2012 [15]. There is a 1-year delay in the reporting of the cause of death compared with the date of death [15]. The date, probable and possible causes of death, and age at death are linked to STHLM0. In Sweden, the death certificates have high reliability of reporting actual prostate cancer-related death [19].
- Prescribed Drug Register.
  This register was established in July 2005 and collects all information of prescribed medications by pharmacies (not during hospitalization) [20]. All pharmacies are obliged to report to the register [21]. Date of dispensing the medication, type of medication, name, strength, pack size, cost, and prescriber are linked to STHLM0.
- NPCR.
  This is a quality register that was established in 1998 and includes all cases of prostate cancer diagnosed in Sweden [8]. It expands on the data available in the Cancer Register and collects clinical information regarding their diagnosis, treatment, and follow-up [8]. It is also linked to other national registers [8]. All information included in the NPCR is linked to STHLM0.
- Other registers and databases.
  STHLM0 is also linked to the microbiology registers and databases, insurance register, histopathological imaging databases, vaccination register, integration database, palliative care, and the Swedish Intensive Care Registry, but these linkages are not updated regularly. In contrast, a linkage to the Education Register and the Multigeneration Register with first-degree male relatives is updated regularly. Diagnostic trials such as STHLM3 and STHLM3-MRI, and studies such as the Stockholm Organized Prostate Cancer Testing (OPT), STHLM1 and 2 have their participants included in STHLM0.
- STHLM0+.
  For comparative studies relating to outcomes of testing and computation of the rates and prevalence, a dataset of all males living in Stockholm County and their first-degree male relatives is available and can be linked to STHLM0. The additional population-based cohort is named STHLM0+, as it supplements STHLM0 and shows the characteristics of those men who did not test for prostate cancer. It included 2 267 103 males as of 31 December 2021.

Table 2 describes the type of data available in STHLM0 and the main registers linked to it. A detailed codebook of the collected variables in STHLM0 is available in Supplementary File 2. From the laboratories that do all Stockholm County’s PSA testing and pathological assessment of biopsies, the total and free PSA is reported and the date of testing. All the pathological reports are retrieved, as detailed in the codebook. Data are available from 2003 onwards, except that data from the Prescribed Drug Register are only available from July 2005 [20, 21].

**Table 2.** Contribution of various registers and databases to the total population of STHLM0 and potential linkage to STHLM0+

Data source	**Number of participants** ^a^	Coverage	Key information
**STHLM0 register**	550 778	2003–ongoing	Detailed below

STHLM0 PSA dataset	549 024	2003–ongoing	Total PSA, free PSA, date of testing
STHLM0 prostate biopsies dataset	66 886	2003–ongoing	Date of biopsy, Gleason grade, SNOMED
Education Register	548 987	2003–ongoing	Education level per year
Swedish Population Register	548 843	2003–ongoing	Civil status per year, income per year, municipality of residence per year, county of residence per year
Swedish Prescribed Drug Register	535 689	2005–ongoing	Medication prescribed, prescription date, package size, dose
Swedish National Patient Register	436 149	2003–ongoing	Admission date, discharge date (for inpatient cases), diagnoses, operations
Cause of Death Register	105 572	2003–ongoing	Date of death, cause of death
National Cancer Register	102 935	2003–ongoing	Date of cancer diagnosis, cancer diagnosis (ICD), SNOMED, TNM staging
National Prostate Cancer Register	41 683	2003–ongoing	Date of diagnosis, diagnostic tests, treatments, date of treatment, pathological findings

**STHLM0+ register**	2 267 103	2003–ongoing	Same datasets as STHLM0

a Number of participants is based on the latest available follow-up of 31 December 2021.

ICD, International Classification of Diseases; PSA, Prostate-specific antigen; SNOMED, Systematized Nomenclature of Medicine; TNM, Tumor, node, metastasis staging system.

Data on PSA results were partially missing between 2003 and 2006 in the south of Stockholm County due to lab loss of data [22]. During that period, only free PSA tests ordered by a physician were recorded [22]. This loss represents ∼15% of the tests done in Stockholm County during that period [22]. Moreover, data on total PSA are recorded in STHLM0 with high completeness, while free PSA tests are not fully reported even after 2006.

### Quality assurance

The new data undergo quality checks before being completely merged with the STHLM0 register. Detailed information on the quality-assurance measures is available in the Supplementary File 1.

## Data resource use

STHLM0 has been used extensively for research, with >30 peer-reviewed original articles published through using this registry. The list of publications that used STHLM0 is available in Supplementary File 1. Some studies relied solely on STHLM0 including its linked registers [9], while others combined data from STHLM0 with other databases and/or cohorts [23].

Overall, STHLM0 provided data that were used in epidemiological studies [9, 10], but also genetic studies [24] and health economics studies [23]. For example, pertaining to screening, Nordström *et al.* found that, despite no recommendations by the Swedish medical guidelines, PSA testing was high and that approximately half of men had a repeat test within 26 months of the initial test [9]. Aly *et al.* found that men with higher PSA (≥10 ng/mL) at diagnosis had a higher risk of recurrence and prostate cancer mortality [25]. Regarding treatment, Vigneswaran *et al.* described that active treatment for castration-resistance metastatic prostate cancer increased from 22% in 2006 to 50% in 2015 [11]. Moreover, Docetaxel was the most-commonly used therapy, while radium-223 was the least-used therapeutic [11]. For one of several health economic evaluations, Du *et al.* showed that artificial intelligence (AI)-assisted workflow (AI+pathologist) would reduce 80% of the biopsy cores viewed by pathologists [26]. Additionally, at a cost of 10 euros per case for AI, the AI-assisted workflow was cost-effective compared with only human pathologists [26].

## Strengths and weaknesses

STHLM0 presents a unique opportunity for researchers who are interested in examining the whole spectrum of the life cycle for men who were tested for prostate cancer. Having access to regularly updated data allows informed decision-making on whether to include them in future screening programs.

STHLM0 has several advantages that are of interest to researchers globally: it includes data from all laboratories that perform prostate cancer diagnostics in Stockholm County—a well-defined population, as the register captures all test results, regardless of outcomes. This makes the register different from the NPCR that has these values at the time of diagnosis of prostate cancer and treatment [27]. Although the Uppsala–Örebro PSA Cohort collects information on men tested using PSA tests since 2005 in Uppsala and Örebro Counties, STHLM0 is the largest register of this kind in Sweden and has the longest follow-up, starting from 2003 [28]. With linkage to expansive and growing list of registers and databases, the potential research scope includes diverse areas that can also support diagnostic studies, clinical studies, and governmental programs for decision-making. With STHLM0+, there is a wider opportunity to study the effects of prostate cancer diagnostics on health outcomes by comparing men who were tested with those who were not. When updates occur, internal quality assurance is performed by the data managers. Additionally, STHLM0 is now updated annually, giving access to new data in a timely fashion. Finally, the steering committee’s [29] diverse expertise enhances collaboration and ensures a systematic, streamlined process.

There are some limitations that STHLM0 faces. Firstly, ∼15% of the PSA test results were not collected due to lab data loss in the south of Stockholm County between 2003 and 2006 [22]. The Swedish Drug Register has initiated their records from July 2005 [21]. Some of the linked datasets were not regularly updated, but there are plans to update the lagged data. Although internal quality-assurance measures are in place, the validity and completeness have not been independently assessed. An assessment of the validity and completeness of the unique variables of STHLM0 is planned in future endeavors.

## Data resource access

Collaborations are welcome. The steering committee [29] meets biannually to approve collaboration plans. Interested researchers are encouraged to submit their collaboration ideas to https://ki.se/en/research/research-areas-centres-and-networks/research-groups/henrik-gronbergs-research-group#tab-sthlm0. Following approval by the steering committee, the research collaborators are expected to get ethics approval from the Swedish Ethics Committee (https://www.etikprovningsansokan.se/). Once the Swedish Ethics Committee approves the project, a data request form can be filled out at the STHLM0 website. For contact e-mail(s), please reach out to Tobias Nordström (tobias.nordstrom@ki.se), Martin Eklund (martin.eklund@ki.se), or Henrik Grönberg (henrik.gronberg@ki.se).

## Ethics approval

STHLM0 was approved by the local ethics committee at Karolinska Institutet and the regional ethics board in Stockholm, with primary application approval number Dnr 2012/438–31/3. All the research conducted using STHLM0 is covered by the approved ethics applications and amendments, and conforms to the Declaration of Helsinki.

## Supplementary Material

dyaf062_Supplementary_Data

## Data Availability

The datasets generated and/or analysed in the current study are not publicly available due to ethical and data-sharing restrictions/laws, including but not limited to the General Data Protection Regulation (GDPR). The data, however, can be requested formally as detailed in the “Data resource access” section. The formal request entails proposal submission, approval of the proposal, acquiring Swedish Ethics Committee approval, and signed research collaboration agreement and data-sharing agreement. Following attainment of the aforementioned steps, the data can be shared.
