# Supplementary material for: Data Resource Profile: STHLM0, the Stockholm Prostate Cancer Diagnostics Register
Source: Int J Epidemiol. 2025 Jun 3;54(3):dyaf062. doi: 10.1093/ije/dyaf062 (PMC12133259; doi:10.1093/ije/dyaf062)
Supplement: dyaf062_Supplementary_Data [file dyaf062_supplementary_data.zip › ije-2024-10-1665-File004.docx]

**Data Resource Profile: STHLM0, the Stockholm Prostate Cancer Diagnostics Register**

**Supplementary File 1: Methods supplement, quality assurance measures, ethical approvals, and list of publications**

**Authors:**

Ahmad Abbadi^1^, Martin Eklund^1^, Markus Aly^2^, Mark Clements^1^, Alessio Crippa^1^, Andrea Discacciati^1^, Astrid Björklund^1^, Vivekananda Lanka^1^, Chiara Micoli^1^, Anna Lantz^1,2^, Henrik Grönberg^1,3^, Tobias Nordström^1,4^

**Affiliations:**

1. Department of Medical Epidemiology and Biostatistics, Karolinska Institutet, Solna, Sweden
2. Department of Molecular Medicine and Surgery (Solna), Karolinska Institutet, Stockholm, Sweden
3. Department of Oncology, Capio St. Görans Sjukhus, Stockholm, Sweden
4. Department of Clinical Sciences at Danderyds Hospital, Karolinska Institutet, Solna, Sweden

**Corresponding author:**

Ahmad Abbadi, MD MMedSc

Department of Medical Epidemiology and Biostatistics, Karolinska Institutet, Solna, Sweden

Nobels väg 12A, 171 65 Solna, Sweden

[ahmad.abbadi@ki.se](mailto:ahmad.abbadi@ki.se)

**Methods:**

This database resource profile utilized data from STHLM0 register, desk-review on the documentation related to STHLM0, and semi-structured interviews with data managers and researchers who upkept STHLM0.

Data from STHLM0 was used with the latest available end of follow-up of 31^st^ December 2021. Descriptive data and figures were presented based on the available data from STHLM0, and showing the number of men each register/dataset is contributing to.

The desk-review was conducted between August 2024-October 2024, where all ethics committee approvals (including amendments) were collected, all previous documentations and main decisions taken on STHLM0 were reviewed, online search on all original papers published using STHLM0, and review of the first two PhD theses that used STHM0 (by Markus Aly, and by Tobias Nordström) was conducted.

Semi-structured interviews were performed, in order of interviews, with Astrid Björklund (data manager), Markus Aly (researcher), Anna Lantz (researcher), Martin Eklund (researcher), Vivekananda Lanka (data manager), Tobias Nordström (researcher), Mark Clements (researcher), and Henrik Grönberg (researcher). The selection of interviewees utilized snowball sampling, with each interviewee being asked if someone should be further interviewed in the process of data collection and assist in the coordination of the interview. Once an interviewee was identified, they were approached by email to request a meeting time, and the purpose of the meeting was disclosed. Verbal consent was collected from all interviewees before the conduct of the interview, and the interviews were recorded. The interviews were then summarized and information was aggregated. Since the interviews did not ask about sensitive information, ethics approval was not needed. Advisory opinion from the ethics committee in Sweden confirmed not needing ethics committee approval in their decision (Dnr 2024-05552-02).

**Data Collected:**

*Quality Assurance Measures:*

The updated datasets undergo quality assurance measures before integration into the previous datasets. The validation rules include automated checks that ascertain if the entries conform to plausible measures of data (e.g., an actual person who is a male and within plausible ages). Moreover, the datasets are compared with previous datasets to ensure no loss of data occurred with the annual update cycle (longitudinal consistency checks), and that the linked data from the registers were fully provided. The cross-checks include comparison to the published information on the linked register and the counts/frequencies relevant. Errors arising in the quality assurance steps are investigated and reported back to SCB for correction, and logs are kept for documentation purposes. The complete raw data is then saved and maintained in a secure file, and version control is performed at each update cycle to ensure previous versions are not lost. Data lags from datasets not updated at each cycle are flagged. Furthermore, access control measures are taken to ensure that researchers are only able to access the datasets relevant to their research, and the person-level datasets are not accessible to any person except for the data manager. As such, only pseudonymized data is available for research use. All researchers are required to conduct data management training before accessing the data, and to have sufficient understanding of the legal and ethical concerns related to handling pseudonymized data. The register is stored at the Department of Medical Epidemiology and Biostatistics at Karolinska Institutet in a secure and encrypted server, and all files are saved in .txt format.

**List of questions used in the semi-structured interview:**

1. Can you describe how STHLM0 was founded?
   1. When?
   2. By whom?
2. Who is considered the team in charge of maintaining STHLM0?
3. What was the main purpose of creating STHLM0?
4. Has the purpose changed or evolved over time?
5. How is it different from the Swedish National Prostate Cancer Register?
   1. Linkage to other registers
   2. Timeline
   3. Specific variables
6. How frequently does data get collected and added to STHLM0?
7. Has there been publications using STHLM0?
   1. When was the first?
8. Has the validity and completeness of STHLM0 been assessed?
9. Where does the funding for STHLM0 come from?
10. What are the main challenges you faced with creating and maintain STHLM0?
11. Do you think the current name “the Stockholm PSA and Biopsy register” is appropriate?
    1. “The Stockholm Prostate Cancer Screening Register”?
    2. Alternatives?
12. As specific as possible, how do you see that STHLM0 can contribute to knowledge in the coming years? Which key action points do you see in order for STHLM0 to be successful in these projects?

*Supplementary Table S1: Ethics committee applications and approvals received for STHLM0 register both in initial work and after official establishment in 2012*

| **Ethics Committee Application No** | **Summary of the application** |
| --- | --- |
| 2008/542-31/2 | Amendment to collect pathology, cytology, clinical chemistry, and immunology outcomes in Stockholm, and to collect all historical data on PSA tests in Stockholm. |
| 2012/438-31/3 | Establishing STHLM0 – collecting all PSA and prostate biopsy tests performed in Stockholm region (Approved March 2012) |
| 2013/101-32 | Adding demographic and socio-economic data from Statistics Sweden. |
| 2013/2088-32 | Authorization to update the database regularly and to save the key file. |
| 2014/460-32 | Add linkage to Swedish Intensive Care Registry (SIR), medical records and authorization to obtain identifiable personal data for linkage and updating to the National Board of Health and Welfare's registers, microbiological registers and the national quality register for prostate cancer. |
| 2014/1877-32 | Permission to review medical records of men whose PSA testing did not lead to follow-up despite indications. |
| 2014/1957/-32 | Extension of the data collection until 2017. |
| 2015/1467-32 | Adding first generation relatives, and access to males living in Stockholm who did not test for prostate cancer. |
| 2016-620-32 | Collect data on alternatives to PSA, such as the Stockholm3 test, the PHI test and the 4K test. |
| 2017/506-32 | Add medical record data on chemotherapy, radiation and X-ray. |
| 2018/248-32 | Sharing the STHLM0 register with other researchers. |
| 2018/568-32 | Enable data sharing with partner organizations. |
| 2018/845-32 | Adding scanned histopathological preparations. |
| 2018/1866-32 | Adding data from palliative care register and operation PAD. |
| 2018/2434-32 | Add link to registers at the Swedish Social Insurance Agency for information on sick leave also for men who do not have prostate cancer. |
| 2019-03155 | Adding linkage to the vaccination register and extension of the preservation of the key for linkage between social security number and serial number at Statistics Sweden until 2025. Original Dnr 2019-05547, mistakenly approved as 2019-03155. |
| 2019-03155 | Add scanned histopathologic specimens and PAD results and allow re-review of prostate biopsies. Note! First Dnr 2019-05547, mistakenly approved as 2019-03155. |
| 2020-00136 | Update the STHLM0 register with MRI data. |
| 2020-01677 | Update the STHLM0 register with data on co-morbidity. |
| 2020-04560 | Adding data from A3P lab, including Stockholm3 test and testosterone level data. |
| 2021-04497 | Adding data for men deceased 2015-2019. |
| 2022-02464-02 | Add link to STATIV database for data on areas. |
| 2024-06808-02 | Including newly established laboratories in Stockholm that perform prostate cancer diagnostics. |
| 2024-08367-02 | Access to the personnummer and electronic medical journals for validation and completion assessment of STHLM0. |
| **Advisory opinions by the ethics committee** | **Summary** |
| 2024-05552-02 | Do not require ethics approval for interviewing data managers and researchers who use STHLM0. |
| 4K: Four-kallikrein, Dnr: Registration/decision number, PHI: Prostate Health Index, PSA: prostate-specific antigen, SIR: Swedish Intensive Care Register, PAD: pathological anatomical diagnosis (pathology report), STATIV: longitudinal database for integration studies, MRI: Magnetic Resonance Imaging | |

**List of Published Peer-reviewed Articles using STHLM0, the Stockholm Prostate Cancer Diagnostics Register**

Updated: 2024-12-31

NB the list only includes published peer-reviewed original papers, and does not consider abstracts, theses, commentaries, books, or other publications.

1. Aly M, Wiklund F, Xu J, Isaacs WB, Eklund M, D'Amato M, Adolfsson J, Grönberg H. Polygenic risk score improves prostate cancer risk prediction: results from the Stockholm-1 cohort study. Eur Urol. 2011 Jul;60(1):21-8. doi: 10.1016/j.eururo.2011.01.017. Epub 2011 Jan 18. PMID: 21295399; PMCID: PMC4417350.
2. Nordström T, Aly M, Clements MS, Weibull CE, Adolfsson J, Grönberg H. Prostate-specific antigen (PSA) testing is prevalent and increasing in Stockholm County, Sweden, Despite no recommendations for PSA screening: results from a population-based study, 2003-2011. Eur Urol. 2013 Mar;63(3):419-25. doi: 10.1016/j.eururo.2012.10.001. Epub 2012 Oct 12. PMID: 23083803.
3. Nordström T, Aly M, Eklund M, Egevad L, Grönberg H. A genetic score can identify men at high risk for prostate cancer among men with prostate-specific antigen of 1-3 ng/ml. Eur Urol. 2014 Jun;65(6):1184-90. doi: 10.1016/j.eururo.2013.07.005. Epub 2013 Jul 19. PMID: 23891454.
4. Nordström, T., Bratt, O., Örtegren, J., Aly, M., Adolfsson, J., & Grönberg, H. (2015). A population-based study on the association between educational length, prostate-specific antigen testing and use of prostate biopsies. Scandinavian Journal of Urology, 50(2), 104–109. https://doi.org/10.3109/21681805.2015.1113200
5. Nordström T, Clements M, Karlsson R, Adolfsson J, Grönberg H. The risk of prostate cancer for men on aspirin, statin or antidiabetic medications. Eur J Cancer. 2015 Apr;51(6):725-33. doi: 10.1016/j.ejca.2015.02.003. Epub 2015 Feb 23. PMID: 25727881.
6. Aly, M., Dyrdak, R., Nordström, T., Jalal, S., Weibull, C.E., Giske, C.G. and Grönberg, H. (2015), Rapid increase in multidrug-resistant enteric bacilli blood stream infection after prostate biopsy—A 10-year population-based cohort study. Prostate, 75: 947-956. https://doi.org/10.1002/pros.22979
7. Nordström T, Vickers A, Assel M, Lilja H, Grönberg H, Eklund M. Comparison Between the Four-kallikrein Panel and Prostate Health Index for Predicting Prostate Cancer. Eur Urol. 2015 Jul;68(1):139-46. doi: 10.1016/j.eururo.2014.08.010. Epub 2014 Aug 20. PMID: 25151013; PMCID: PMC4503229.
8. Wallerstedt A, Strom P, Gronberg H, Nordstrom T, Eklund M. Risk of Prostate Cancer in Men Treated With 5α-Reductase Inhibitors-A Large Population-Based Prospective Study. J Natl Cancer Inst. 2018 Nov 1;110(11):1216-1221. doi: 10.1093/jnci/djy036. PMID: 29548030.
9. Aly M, Clements M, Weibull CE, Nordström T, Näslund E, Adolfsson J, Grönberg H. Poor Follow-up After Elevated Prostate-specific Antigen Tests: A Population-based Cohort Study. Eur Urol Focus. 2019 Sep;5(5):842-848. doi: 10.1016/j.euf.2018.02.001. Epub 2018 Feb 9. PMID: 29433987.
10. Karlsson A, Jauhiainen A, Gulati R, Eklund M, Grönberg H, Etzioni R, Clements M. A natural history model for planning prostate cancer testing: Calibration and validation using Swedish registry data. PLoS One. 2019 Feb 14;14(2). doi: 10.1371/journal.pone.0211918. PMID: 30763406; PMCID: PMC6375591.
11. Palsdottir T, Nordstrom T, Karlsson A, et al. The impact of different prostate-specific antigen (PSA) testing intervals on Gleason score at diagnosis and the risk of experiencing false-positive biopsy recommendations: a population-based cohort study BMJ Open 2019;9. doi: 10.1136/bmjopen-2018-027958
12. Beckmann K, Kinsella N, Olsson H, Wallerstedt Lantz A, Nordstrom T, Aly M, Adolfsson J, Eklund M, Van Hemelrijck M. Is there any association between prostate-specific antigen screening frequency and uptake of active surveillance in men with low or very low risk prostate cancer? BMC Urol. 2019 Aug 5;19(1):73. doi: 10.1186/s12894-019-0502-4. PMID: 31383015; PMCID: PMC6683376.
13. Beckmann K, Crawley D, Nordström T, Aly M, Olsson H, Lantz A, Binti Abd Jalal N, Garmo H, Adolfsson J, Eklund M, Van Hemelrijck M. Association Between Antidiabetic Medications and Prostate-Specific Antigen Levels and Biopsy Results. JAMA Netw Open. 2019 Nov 1;2(11). doi: 10.1001/jamanetworkopen.2019.14689. PMID: 31693126; PMCID: PMC6865613.
14. Hao S, Östensson E, Eklund M, Grönberg H, Nordström T, Heintz E, Clements M. The economic burden of prostate cancer - a Swedish prevalence-based register study. BMC Health Serv Res. 2020 May 20;20(1):448. doi: 10.1186/s12913-020-05265-8. PMID: 32434566; PMCID: PMC7238534.
15. Aly, M., Leval, A., Schain, F., Liwing, J., Lawson, J., Vágó, E., … Akre, O. (2020). Survival in patients diagnosed with castration-resistant prostate cancer: a population-based observational study in Sweden. Scandinavian Journal of Urology, 54(2), 115–121. https://doi.org/10.1080/21681805.2020.1739139
16. Ström P, Nordström T, Delahunt B, Samaratunga H, Grönberg H, Egevad L, Eklund M. Prognostic value of perineural invasion in prostate needle biopsies: a population-based study of patients treated by radical prostatectomy. J Clin Pathol. 2020 Oct;73(10):630-635. doi: 10.1136/jclinpath-2019-206300. Epub 2020 Feb 7. PMID: 32034057; PMCID: PMC7513266.
17. Björnebo, L., Olsson, H., Nordström, T. et al. Predictors of adverse pathology on radical prostatectomy specimen in men initially enrolled in active surveillance for low-risk prostate cancer. World J Urol 39, 1797–1804 (2021). <https://doi.org/10.1007/s00345-020-03394-7>
18. Vigneswaran, H. T., Warnqvist, A., Andersson, T. M. L., Leval, A., Eklund, M., Nordström, T., … Aly, M. (2021). Real world treatment utilization patterns in patients with castration-resistant prostate cancer. *Scandinavian Journal of Urology*, *55*(4), 299–306. https://doi.org/10.1080/21681805.2021.1936626
19. Karlsson AA, Hao S, Jauhiainen A, Elfström KM, Egevad L, Nordström T, et al. (2021) The cost-effectiveness of prostate cancer screening using the Stockholm3 test. PLoS ONE 16(2): e0246674. https://doi.org/10.1371/journal.pone.0246674
20. Khoshkar Y, Westerberg M, Adolfsson J, Bill-Axelson A, Olsson H, Eklund M, Akre O, Garmo H, Aly M. Mortality in men with castration-resistant prostate cancer-A long-term follow-up of a population-based real-world cohort. BJUI Compass. 2021 Oct 10;3(2):173-183. doi: 10.1002/bco2.116. PMID: 35474724; PMCID: PMC8988790.
21. Khoshkar, Y., Vigneswaran, H. T., Eloranta, S., Andersson, T. M. L., Schain, F., Boman, A., … Aly, M. (2022). Cardiovascular, bone, and metabolic health in men with castrate-resistant prostate cancer treated with androgen deprivation: a matched cohort study. Acta Oncologica, 61(11), 1377–1385. <https://doi.org/10.1080/0284186X.2022.2141077>
22. Ventimiglia E, Bill-Axelson A, Adolfsson J, Aly M, Eklund M, Westerberg M, Stattin P, Garmo H. Modeling Disease Trajectories for Castration-resistant Prostate Cancer Using Nationwide Population-based Data. Eur Urol Open Sci. 2022 Aug 23;44:46-51. doi: 10.1016/j.euros.2022.07.010. PMID: 36185582; PMCID: PMC9520495.
23. Björnebo L, Nordström T, Discacciati A, Palsdottir T, Aly M, Grönberg H, Eklund M, Lantz A. Association of 5α-Reductase Inhibitors With Prostate Cancer Mortality. JAMA Oncol. 2022 Jul 1;8(7):1019-1026. doi: 10.1001/jamaoncol.2022.1501. Erratum in: JAMA Oncol. 2022 Jun 16. doi: 10.1001/jamaoncol.2022.2696. Erratum in: JAMA Oncol. 2022 Oct 1;8(10):1518. doi: 10.1001/jamaoncol.2022.3737. PMID: 35587340; PMCID: PMC9121300.
24. Hao S, Heintz E, Östensson E, Discacciati A, Jäderling F, Grönberg H, Eklund M, Nordström T, Clements MS. Cost-Effectiveness of the Stockholm3 Test and Magnetic Resonance Imaging in Prostate Cancer Screening: A Microsimulation Study. Eur Urol. 2022 Jul;82(1):12-19. doi: 10.1016/j.eururo.2021.12.021. Epub 2022 Jan 31. Erratum in: Eur Urol. 2022 Jul;82(1). doi: 10.1016/j.eururo.2022.03.025. PMID: 35094896.
25. Bonde TM, Westerberg M, Aly M, Eklund M, Adolfsson J, Bill-Axelson A, Garmo H, Stattin P, Robinson D. Time to castration-resistant prostate cancer and prostate cancer death according to PSA response in men with non-metastatic prostate cancer treated with gonadotropin releasing hormone agonists. Scand J Urol. 2022 Jun;56(3):169-175. doi: 10.1080/21681805.2022.2070275. Epub 2022 May 12. PMID: 35548951.
26. Keeney, E., Sanghera, S., Martin, R.M. *et al.* Cost-Effectiveness Analysis of Prostate Cancer Screening in the UK: A Decision Model Analysis Based on the CAP Trial. *PharmacoEconomics* **40**, 1207–1220 (2022). https://doi.org/10.1007/s40273-022-01191-1
27. Hao S, Discacciati A, Eklund M, et al. Cost-effectiveness of Prostate Cancer Screening Using Magnetic Resonance Imaging or Standard Biopsy Based on the STHLM3-MRI Study. JAMA Oncol. 2023;9(1):88–94. doi:10.1001/jamaoncol.2022.5252
28. Falagario UG, Abbadi A, Remmers S, et al. Biochemical Recurrence and Risk of Mortality Following Radiotherapy or Radical Prostatectomy. *JAMA Netw Open.* 2023;6(9):e2332900. doi:10.1001/jamanetworkopen.2023.32900
29. Arvendell M, Björnebo L, Eklund M, Giovanni Falagario U, Chandra Engel J, Akre O, Grönberg H, Nordström T, Lantz A. Prediagnostic Prostate-specific Antigen Testing and Clinical Characteristics in Men with Lethal Prostate Cancer. Eur Urol Open Sci. 2024 Mar 4;62:61-67. doi: 10.1016/j.euros.2024.02.011. PMID: 38468863; PMCID: PMC10925930.
30. Du X, Hao S, Olsson H, Kartasalo K, Mulliqi N, Rai B, Menges D, Heintz E, Egevad L, Eklund M, Clements M. Effectiveness and Cost-effectiveness of Artificial Intelligence-assisted Pathology for Prostate Cancer Diagnosis in Sweden: A Microsimulation Study. Eur Urol Oncol. 2024 May 23:S2588-9311(24)00133-0. doi: 10.1016/j.euo.2024.05.004. Epub ahead of print. PMID: 38789385.
31. Rai B, Nordström T, Lantz A, Lund RL, Kuja-Halkola R, Rado M, Öberg S, Hao S, Du X, Clements M. Spatio-temporal variation in prostate cancer testing in Stockholm: A population-based study. PLoS One. 2024 Aug 15;19(8):e0308254. doi: 10.1371/journal.pone.0308254. PMID: 39146336; PMCID: PMC11326630.
32. Björnebo L, Razdan S, Discacciati A, Palsdottir T, Aly M, Nordström T, Eklund M, Lundon D, Grönberg H, Tewari A, Wiklund P, Kyprianou N, Lantz A. Prostate cancer incidence and mortality in men exposed to α1-adrenergic receptor antagonists. J Natl Cancer Inst. 2024 Sep 1;116(9):1459-1465. doi: 10.1093/jnci/djae108. PMID: 38718219; PMCID: PMC11378311.
